# Enhancement of docosahexaenoic acid (DHA) production from *Schizochytrium* sp. S31 using different growth medium conditions

**DOI:** 10.1186/s13568-018-0540-4

**Published:** 2018-01-24

**Authors:** Deniz Sahin, Ezgi Tas, Ulkü Hüma Altindag

**Affiliations:** Department of Molecular Biology and Genetics, Istanbul Technical University, Maslak, 34469 Istanbul, Turkey

**Keywords:** DHA, Omega-3, Polyunsaturated fatty acids, *Schizochytrium* sp.

## Abstract

*Schizochytrium* species is one of the most studied microalgae for production of docosahexaenoic acid (DHA) which is an omega-3 fatty acid with positive effects for human health. However, high cost and low yield in production phase makes optimization of cultivation process inevitable. We focus on the optimization of DHA production using *Schizochytrium* sp. using different media supplements; glucose, fructose and glycerol as carbon variants, proteose peptone and tryptone as nitrogen variants. The highest biomass (5.61 g/L) and total fatty acid yield (1.74 g/L) were obtained in proteose peptone medium which was used as the alternative nitrogen source instead of yeast extract. The highest DHA yield (0.40 g/L) was achieved with glycerol as the carbon source although it had the second lowest biomass production after ethanol containing medium. Ethanol, as an alternative carbon source and a precursor for acetyl-CoA, increased DHA percentage in total lipid content from 29.94 to 40.04% but decreasing the biomass drastically. Considering different carbon and nitrogen sources during cultivation of *Schizochytrium* sp. will improve DHA production. Combination of proteose peptone and glycerol as nitrogen and carbon sources, respectively, and addition of ethanol with a proper timing will be useful to have higher DHA yield.

**Electronic supplementary material:**

The online version of this article (10.1186/s13568-018-0540-4) contains supplementary material, which is available to authorized users.

## Introduction

Long chain polyunsaturated fatty acids (LC-PUFAs) are known as crucial for human nutrition with their roles in the development of human brain in early stages of life. Omega-3 fatty acids, particularly DHA and EPA (Eicosapentaenoic acid), are of the importance for human development between all LC-PUFAs. DHA (C22:6 ω-3) has been shown to contribute the enhancement of human brain in the course of evolution along with mediating various metabolisms such as development of normal composition of sperm retina and brain lipids (Crawford et al. 1999). In the absence of DHA, abnormalities in brain function has been demonstrated (Tapiero et al. 2002). Moreover, high levels of DHA intake through diet affects amyloid precursor protein processing and prevents β-amyloid production thereby lower the risk of occurrence of Alzheimer disease (Lim et al. 2005). Omega-3 fatty acids are important to keep maintain the healthy state of immune system suggesting a new approach for autoimmune diseases, as well as cardiovascular system and nervous system disorders (Mehta et al. 1988; Simopoulos 2002).

Dietary supplements for humans that are rich in DHA covers a large share of the market and are mostly produced from fish rather than microbial sources (Martins et al. 2013). However, the fish stocks used for oil production have reached to extend (Shene et al. 2010). Thus, the production of these omega-3 rich oils demands new sources and the efforts in developing microbial production can increase the market share by reducing the prices. The algal sources have their advantages as being consumers of carbon dioxide and growing in salt water (Martins et al. 2013). PUFA yield depends on environmental conditions such as nitrate starvation, increased salinity changed concentrations of carbon and nitrogen composition and their sources, and variations at light intensity (Yokochi et al. 1998). The photosynthetic production of n-3 LC-PUFA is not profitable due to low biomass density resulting in low production of PUFAs. Main routes for improving the n-3 PUFAs are increasing the content of desired lipids per units of biomass and increasing the biomass density to enhance the biomass per culture volume. During the optimization of cell growth, the challenging part is that LC-PUFAs are generated under abiotic stress which results in a decrease in the biomass yield. In the future aspect the research can be driven to find an alternative sustainable pathway for PUFA accumulation.

*Schizochytrium* strains can be cultivated with different sugars such as glucose, fructose, mannose, and galactose as carbon sources. Physiological conditions affect the yields of biomass and fatty acid composition of cultivated cells along with the composition of the medium (Patil and Gogate 2015). *Schizochytrium* is known for its bulging growth rate and enhanced capability to produce DHA quantity when grown on glucose or fructose (Chatdumrong et al. 2007). Excess of C and limiting N in medium usually result in lipid accumulation, however, low amount of N causes reduction in cell growth which give rise to lower lipid and DHA yield. Glucose, dl-malic acid, d-fructose, d-xylose and glycerol have been used as carbon source with successive cell growth and DHA yield of 20% in the biomass. In contrast, di- and polysaccharides caused limited cell growth (Shene et al. 2010). In a study, comparison of glucose, fructose and glycerol as carbon source give the result of 32.5, 30.9 and 43.1% DHA in fatty acid composition (Yokochi et al. 1998). The initial pH of medium alter the DHA yield and total lipid accumulation by affecting cell membrane function and the uptake of nutrients. The maximum DHA yield and biomass of *S. limacinum* has been achieved at pH 7 (Wu et al. 2005). Other determinant is the salinity which regulates the cytoplasmic ion gradient and activity of enzymes (Kim et al. 2005). According to a study, in which different seawater concentrations were used, lowering the salinity from 28 to 18% resulted in higher DHA accumulation (Zhu et al. 2008).

Here, DHA production in *Schizochytrium* sp. was tried to be optimized by using alternative media components. Different carbon and nitrogen sources were tested to find the highest biomass and DHA yield. Moreover, the effect of ethanol addition as an alternative carbon source and a precursor for acetyl-CoA, at the lipid accumulation stage was investigated.

The highest cell growth, total fatty acid, and DHA yield were achieved with proteose peptone medium as a nitrogen variant. On the other hand, CM medium with glucose as carbon source presented the highest biomass compared to FM and GM media containing fructose and glycerol, respectively as carbon sources. GM medium with glycerol had the lowest biomass among alternative carbon sources. However, lipid analysis showed that the highest DHA yield among tested carbon sources was achieved with GM medium with glycerol as the carbon source.

## Materials and methods

### Microorganism and initial growth medium

The *Schizochytrium* sp. S31 was obtained from American Type Culture Collection (ATCC^®^ 20888™), stored as frozen cultures and cultivated in complex medium (CM) containing glucose (40 g/L), proteose peptone (8 g/L), yeast extract (5 g/L), NaCl (25 g/L) and MOPS (21 g/L) in 100 mL baffled flask. Cultures were then scaled up to 2 L Erlenmeyer flask and incubated at 27 °C on a shaker (200 rpm). When the starting culture reached absorbance value of 2.4 at 660 nm, 1:100 (w/w) ratio of the culture was used for scaling up (400 mL medium in 2 L flasks).

### Cultivation in different growth mediums

*Schizochytrium* cells were cultivated in different growth mediums with changing carbon and nitrogen sources. CM medium was taken as the control medium. Following mediums were prepared by changing C or N sources in CM while keeping other contents constant.

Fructose medium (FM) had 40 g/L fructose instead of glucose in CM. Glycerol medium (GM) contained 3.24:1000 (v/v) ratio of glycerol as carbon source. Tryptone medium (TM) contained 5 g/L tryptone instead of yeast extract. For the proteose peptone medium (PPM), yeast extract was not included in the medium, instead total proteose peptone concentration was increased to 13 g/L. To check the effect of ethanol addition on DHA and biomass production, cells were cultivated in CM medium and 40 mL/L ethanol was added at the 24th hours of incubation.

*Schizochytrium* cells were cultivated in changing growth mediums for 144 h in 2 L flasks at 27 °C on a shaker (200 rpm). Every 24 h, samples were collected from each medium to check the cell biomass and absorbance at OD_600_. In addition, the pH values were measured during the incubation period. At the end of 144th hours, all cells were recovered by centrifugation at 5000 rpm for 5 min.

### Cell disruption and lipid extraction

After the centrifugation, the cells were washed with distilled water and freeze dried for 24 h to eliminate the water from the cells. After freeze-drying process, dry cell biomass for each sample were measured and recorded. Dried cell pellets were grinded until they gained the powder form and then transferred to falcon tubes. For the lipid extraction process, *n*-hexane solution (Sigma-Aldrich) was added to samples in 6:1 ratio (v/w). The cells were sonicated (three bursts of 20 s each) for further disruption of the samples. Then, falcon tubes were placed on a shaker at 150 rpm and incubated for 6 h at 27 °C. After incubation, the cells were centrifuged at 5000 rpm for 5 min and supernatants were transferred into clean tubes. Volatilization step was done under fume hood and it continued until a viscous liquid was formed at the bottom falcon tubes. The weight and volume of lipids were calculated. Then, fatty acid composition of each extracted lipid samples were determined by using GC-FID (Agilent Technologies 6890 N), gas chromatography with flame ionization detector (A&T Gida Kontrol Laboratuvarı, Istanbul).

### Fatty acid composition analysis

Fatty acid methyl esters (FAME) were prepared by a modified standard method as follows (Veeneman 2011): samples that were dissolved in *n*-hexane processed further with cold esterification method. 2 N Potassium hydroxide/methanol was added to extracted oil samples. Then it was centrifuged at 4000 rpm for 10 min. The trans-esterified FAMEs were extracted into the upper *n*-hexane layer. Gas chromatography (GC) analysis was performed by injecting *n*-hexane layer separately (30/1) into gas Chromatography device (flame ionization detector, GC-FID).

GC (Agilent Technologies 6890 N) analysis conditions were as follows: A capillary column (Agilent HP-88 with sizes 100 mm × 0.25 mm × 0.2 µm) was applied; Oven temperature was kept at 140 °C for 5 min then increased till 240 °C at 4 °C/min and was maintained in this temperature for 30 min. The injection was completed in total of 60 min. Inlet and detector (FID) temperatures were set at 250–280 °C respectively. Helium was used as carrier gas. Fatty acids were identified by comparison to peaks of external standards (Supelco FAME-MIX with 37 components) and the amount of FAMEs in samples were quantified.

## Results

### Growth curve of *Schizochytrium* sp.

**Fig. 1:**
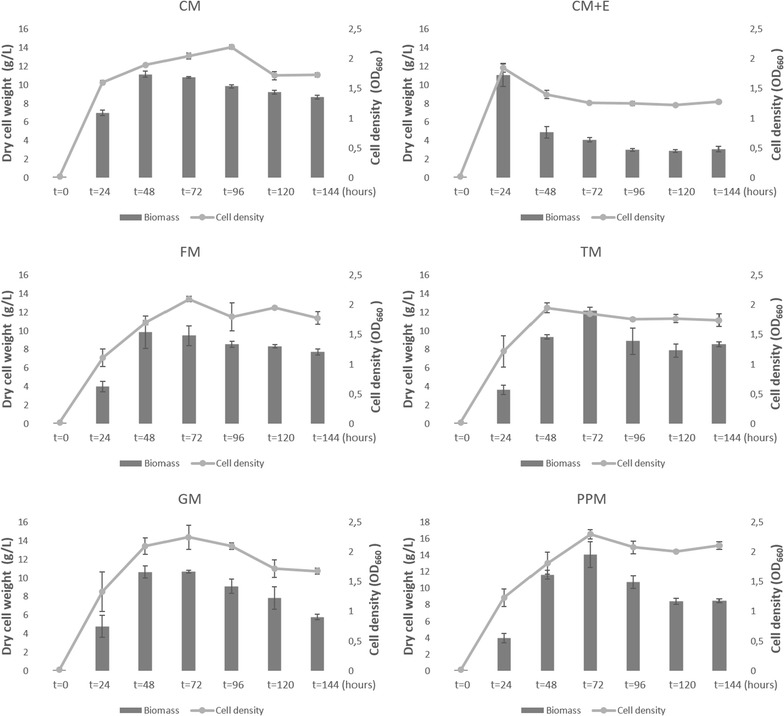
Growth curve of *Schizochytrium* sp. in different mediums: *Schizochytrium* sp. were grown in CM, CM + E, FM, TM, GM, PPM mediums with total 144 h incubation. The highest cell biomass during incubation was achieved at 72th hours in TM, GM, PPM while in CM and FM it was achieved at 48th hours. CM + E has shown lower biomass accumulation pattern in comparison with other mediums. The cell density pattern in FM, GM and PPM has shown highest value at 72th hours and TM has shown the very near results at 48 and 72th hours for the highest cell density. In CM, highest cell density was achieved at 96th hours. In CM + E cell density has not changed significantly after introduction of ethanol at 24th hours and it was rather lower than other mediums

*Schizochytrium* sp. was incubated in media varying with carbon and nitrogen sources and OD_660_ was measured by spectrophotometer in a 6 days period. Figure 1 shows cell densities along with the cell dry weight change throughout the incubation time for each growth medium. Glucose (in CM medium), ethanol, fructose and glycerol were used as alternative carbon sources. Tryptone and proteose peptone were used as alternative nitrogen sources instead of yeast extract in the medium. In CM medium, logarithmic phase started to slow down between the 48th and 72nd hours as OD_660_ values indicated. The highest biomass was achieved as 11.15 g/L at the 48th hours while highest cell density was observed at the 96th as 2.2. In the 2nd CM medium, addition of ethanol after the 24th hours decreased the cell density until the 72nd hours and remained stable until the end of the measurement. The highest biomass and cell density were achieved at the 24th hours which was before the addition of ethanol and yielded as 11.05 g/L and 1.9, respectively. Cells cultivated in FM, GM and PPM media showed log phase indicators till the 72nd hours and they entered to the stationary phase. The highest cell biomass and cell density values were obtained at the 72th hours for FM, GM and PPM as 9.5, 10.7, 14.1 g/L and 2.1, 2.25 and 2.3 (OD_660_), respectively. The log phase in TM medium continued until the 48th hour and reached highest cell density as 1.95, however it achieved the highest cell biomass at 72th hour as 12.2 g/L. The highest cell dry weight and highest absorbance values are mostly correlated with each other.

### Total biomass, total fatty acids and DHA yield determination

**Fig. 2:**
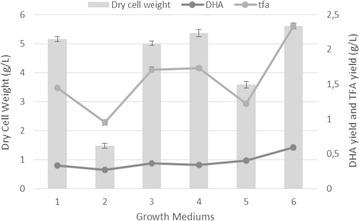
Dry cell weight, Total fatty acid yield and DHA yields in CM (1), CM + E (2), FM (3), TM (4), GM (5) and PPM (6) media

Figure 2 shows the total dry cell weight, total fatty acid amount and calculated amount of DHA based on fatty acid composition percentages obtained from GC-FID analysis at the end of 144th hours. DHA yield determination was carried out by multiplying GC-FID fatty acid composition percentages of DHA to total dry cell weight. The highest cell growth was observed in PPM medium (5.611 g/L) which had proteose peptone as nitrogen source instead of yeast extract while CM medium had lower cell growth (5.15 g/L). TM medium had the second highest cell growth (5.36 g/L), which is still higher than the CM medium.

Fructose and glycerol were used as carbon sources alternative to glucose to compare the effects of C source on cell growth, biomass production and fatty acid composition. Among all, glucose in CM medium showed the highest biomass (5.15 g/L), fructose as the second (5.01 g/L) and glycerol as the lowest biomass (3.58 g/L) production at the end of 144th hours. However, the striking result was observed with the lipid analysis. The highest DHA yield (0.40 g/L) among tested carbon sources achieved with glycerol that has the lowest cell biomass.

### pH variations

**Fig. 3:**
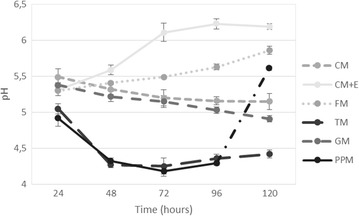
pH variation with time for each medium

Figure 3 indicates the pH variation for each media with different carbon and nitrogen sources. CM medium started with pH 5.49 which was relatively high compared to the others. PPM medium started with pH 4.92. For CM + E medium, pH started to increase gradually from 5.31 to 6.23 after the addition of ethanol. The other significant variation was observed in GM medium with its pH decrease to 4.91.

### Fatty acid composition

Table 1 shows the fatty acid compositions of the samples at the 144th hours cultivated in different media. The highest DHA percentage (48.75%) was achieved with GM medium while percentages of palmitic acid (C16:0) and mostly pentadecanoic acid (C15:0) decreased compared to the CM medium.

**Table 1:** Fatty acid compositions (%:w/v) according to Gas chromatography-FID analysis of obtained total fatty acids (Additional file 1)

	CM	CM + E	FM	TM	GM	PPM
Omega-3						
Docosahexaenoic acid (C22:6 n-3)	29.94	40.04	26.53	24.81	48.75	34.13
Eicosapentaenoic acid (C20:5 n-3)	1.48	6.05	1.54	1.22	3.8	1.71
Linolenic acid (C18:3 n-3)	0.07	–	0.07	0.05	0.08	0.05
Omega-6						
γ-Linolenic acid (C18:3 n-6)	0.2	0.31	0.23	0.17	0.21	0.19
Arachidonic acid (C20:4 n-6)	0.61	2.73	0.66	0.51	1.69	0.71
*cis*-8,11,14-Eicosatrienoic acid (C20:3 n-6)	0.35	0.11	–	0.31	0.41	0.35
Others						
Pentadecanoic acid (C15:0)	28.67	18.47	26.79	29.71	14.4	22.63
Palmitic acid (C16:0)	20.19	15.81	24.38	22.79	17.81	21.4
Margaric acid (C17:0)	9.76	6.14	9.28	8.02	6.74	8.32
Myristic acid (C14:0)	4.79	3.19	6.48	5.58	2.47	4.01
Stearic acid (C18:0)	1.61	3.92	1.59	1.41	2.26	1.37
Tridecanoic acid (C13:0)	1.57	1.33	1.57	1.87	0.56	1.04
Lauric acid (C12:0)	0.25	0.23	0.39	0.23	0.11	0.18
Undecanoic acid (C11:0)	0.13	–	0.12	0.14	0.07	0.09
Oleic acid (C11:1n + 9c)	0.09	0.2	0.05	0.04	0.1	0.01
Palmitoleic acid (C16:1)	0.09	0.96	0.15	0.13	0.15	0.08
Lignoceric acid (C24:0)	0.07	0.36	0.08	0.12	0.1	0.13
Arachidic acid (C20:0)	0.03	0.14	0.03	0.04	0.04	0.04
Behenic acid (C22:0)	0.03	–	–	–	0.09	0.03
γ-Linoleic acid (C18:2n + 6c)	0.02	–	0.02	0.01	0.04	0.01
Heneicosanoic acid (C21:0)	0.01	–	0.02	0.01	0.05	0.29
Heptadecenoic acid (C17:1)	0.01	–	0.01	–	–	–
Tricosanoic acid (C23:0)	–	–	–	2.83	0.07	3.22

*CM* complex medium, *FM* fructose medium, *TM* tryptone medium, *GM* glycerol medium, *PPM* proteose peptone medium

The second highest DHA percentage (40.04%) was observed in CM + E medium, however, the cell biomass was the lowest among all (1.48 g/L), as it is indicated in Fig. 2.

In PPM medium, the percentage of DHA is 34.13%, which is higher than the CM medium. Although it was not the highest DHA percentage, the highest DHA yield (0.59 g/L) was achieved in PPM medium since total lipid production was the highest (Table 2).

**Table 2:** Overall comparison of total biomass production and DHA yields and EPA yields for all media

	Total biomass (g/L)	DHA yield (g/L)	EPA yield (g/L)	References
CM	5.15 (± 0.09)	0.33 (± 0.005)	0.016 (± 0.0005)	This study
CM + E	1.48 (± 0.09)	0.27 (± 0.015)	0.041 (± 0.0025)	This study
FM	5.01 (± 0.07)	0.36 (± 0.01)	0.021 (± 0.00065)	This study
TM	5.36 (± 0.12)	0.34 (± 0.005)	0.017 (± 0.00035)	This study
GM	3.58 (± 0.12)	0.40 (± 0.015)	0.031 (± 0.001)	This study
PPM	5.61 (± 0.1)	0.59 (± 0.01)	0.029 (± 0.0005)	This study
CM	5.510	0.308	–	Wu et al. (2005)
FM	5.245	0.251	–	Wu et al. (2005)
TM	2.133	0.178	–	Wu et al. (2005)
PPM	5.220	0.084	–	Wu et al. (2005)
CM	8.78 ± 0.55	0.51 ± 0.04	–	Zhu et al. (2008)
FM	9.02 ± 0.38	0.34 ± 0.02	–	Zhu et al. (2008)
TM	11.07 ± 0.52	0.79 ± 0.11	–	Zhu et al. (2008)
PPM	8.34 ± 0.42	0.23 ± 0.03	–	Zhu et al. (2008)
GM	12.18 ± 0.47	0.68 ± 0.06	–	Zhu et al. (2008)
CM	~ 15.1	~ 0.7	~ 0.00184	Yokochi et al. (1998)
FM	~ 13.66	~ 0.93	~ 0.00666	Yokochi et al. (1998)
TM	~ 3.3	~ 0.34	Trace	Yokochi et al. (1998)
GM	~ 15.1	~ 1.33	~ 0.021	Yokochi et al. (1998)

*CM* complex medium, *FM* fructose medium, *TM* tryptone medium, *GM* glycerol medium, *PPM* proteose peptone medium

In FM and TM media, the percentage of DHA (26.53 and 24.81%) in lipid solution were lower than the CM medium (29.94%), however, DHA yields were similar (0.36, 0.34, 0.33 g/L, FM, TM, CM respectively) thanks to the higher lipid accumulation in FM and TM. In spite of the highest DHA percentage in GM, the second highest DHA yield could be achieved with this media due to the lower biomass accumulation than PPM (3.58, 5.61 g/L for GM and PPM respectively).

## Discussion

Here we used different media supplements as carbon and nitrogen variants for the optimization of DHA production in *Schizochytrium* species. Figure 1 shows the change in biomass and cell densities throughout the incubation time for each growth medium. The growth curves were varied for each media and lag phase, for each sample, was not observed likely due to the time gaps between two measurements in which the lag phase has already proceeded. Biomass values were related to each other according to the time they entered the stationary phase. At the end of 144th hours, total dry cell weight, total fatty acid amount and DHA amount were calculated as given in Fig. 2. The results are correlated with the literature except for peptone, in a study the cell biomass reached its peak with tryptone then followed by yeast extract and peptone as second and third respectively (Zhu et al. 2008). The total nitrogen content of each nitrogen source can explain the difference between biomasses. Yeast extract has 10.5% total nitrogen whereas proteose peptone has 12% according to product information sheets. Based on the results of this study, proteose peptone can be used as nitrogen source to obtain high cell growth. The effect of nitrogen source can be evaluated according to the data shown in Fig. 2. DHA yield and lipid accumulation are proportional with the cell biomass. The highest cell growth (5.611 g/L), total fatty acid (1.74 g/L) and DHA yield (0.59 g/L) were achieved with proteose peptone medium.

Yokochi et al. (1998) tested glucose, fructose and glycerol as carbon sources and also showed the similar results with respect to the biomass production, highest in glucose, then fructose, and glycerol. According to the DHA yields, the study still correlates with the fact that highest yield of DHA was obtained in glycerol cultivation medium.

Lipid accumulation is achieved in microorganisms relying on two conditions; continuous supply of acetyl CoA in the cytosol as precursor and continuous supply of NADPH as the required reductant in fatty acid biosynthesis (Botham and Ratledge 1979). Acetyl CoA can be added directly to the cytosol of cells. On the other hand, ethanol can be used as alternative carbon source for DHA production since it can easily be converted into acetyl-CoA in eukaryotes. The advantage of using ethanol is its low cost and availability compared to acetyl CoA. Ethanol addition, which decreases the amount of nitrogen, results with the excess of carbon. Therefore, the cell enters a phase called rapid lipid accumulation state which usually begins after 40 h of cell growth, and in this phase biomass of the cell remains constant with lipid accumulation in low amounts (Hawley and Gordon 1976). In this study ethanol (40 mL/L final concentration) was added to the growth medium at the 24th hours which is the assumed late lipid accumulation stage. In a previous study, the addition of 40 mL/L ethanol has resulted in slight biomass reduction while increasing DHA percentage from 35 to 38% (Zhu et al. 2008). In this study, percentage of DHA was increased from 29.94 to 40.04%. On the other hand, the biomass of ethanol-added medium was measured as 1.48 g/L (biomass for CM is 5.15 g/L). This means 71.26% decrease in biomass that can be explained with the toxicity of ethanol towards the cells. However, according to lipid analysis, yield of DHA reduced by 19.14%.

All of the media except CM + E and FM media followed a decreasing trend of pH. Wu et al. (2005) has reported that initial pH of the cultivation medium along with the carbon and nitrogen sources affect the DHA yield in a combinatorial way. Highest DHA yield was achieved near the neutral pH. However, growth or lipid production has not occurred above pH 7. Here, we observed that the highest DHA yield was observed in PPM and CM + E media and the pH values were also accorded with that evaluation. According to the same study (Wu et al. 2005), the gradual reduction can be explained with the secretion of organic acids such as succinic acid, pyruvic acid, and malic acid but their amounts were also determined with the initial pH values.

GM medium has the highest DHA percentage in fatty acid composition analysis as given in Table 1. As DHA percentage increases, palmitic acid (C16:0) and mostly pentadecanoic acid (C15:0) decrease compared to the CM medium. They are both saturated fatty acids. Based on a previous study, high amount of palmitic acid causes inhibition of chemotaxis and phagocytosis which will affect the functions of immune system cells (Hawley and Gordon 1976). Pentadecanoic acid has been used as a biomarker for the detection of milk fat through diet since rumen microbiota and microbial de-novo lipogenesis produces high levels of pentadecanoic acid (Jenkins et al. 2015). Decrease in this saturated fatty acids may be an indicator of a metabolic pathway that results in DHA production under the effect of glycerol as carbon source.

Although CM + E medium has the second highest DHA percentage (40.04%), the cell biomass was the lowest among all (1.48 g/L), as it is indicated in Fig. 2. The effects of ethanol and late lipid accumulation stage can be observed significantly. There is a decrease in pentadecanoic acid and palmitic acid percentage, as well.

Even though, the aim of this study was not to optimize the EPA production, the yield of EPA can be seen in Table 2 which also lists total biomass, DHA and EPA yields from this study and other studies. Yields of EPA was not significantly high in each medium, however, in CM + E, the highest EPA yield was obtained (0.041 g/L) with highest EPA percentage of 6.05% among all mediums. In CM, TM, FM, the yield of EPA was very similar 0.016, 0.017 and 0.021 g/L. GM and PPM has shown similar EPA yield as 0.031 and 0.029 g/L, respectively.

*Schizochytrium* sp. is one of the most studied alternative producer organism for omega-3 fatty acids, specifically docosahexaenoic acid-DHA. Changing carbon and nitrogen sources in the cultivation medium will affect the biomass, fatty acid and DHA production. Here, different media supplements; glucose, fructose and glycerol as carbon variants, proteose peptone and tryptone as nitrogen variants, were tried to enhance the DHA production. Overall, the highest biomass and yield were achieved with proteose peptone as sole nitrogen source. Glycerol was the best choice to have higher yield even with lower biomass production. Addition of ethanol enhances the DHA production but yield is low because of decreased biomass production. Combination of proteose peptone as nitrogen source and glycerol as carbon source, and addition of ethanol with a proper timing will be useful to have better DHA yield.

## Additional file

**Additional file 1: Figure S1.** GC-FID spectra and area percent report for fatty acid extract from *Schizochytrium* sp. 31. in CM medium.** Figure S2.** GC-FID spectra and area percent report for fatty acid extract from *Schizochytrium* sp. 31. in CM+E medium.** Figure S3.** GC-FID spectra and area percent report for fatty acid extract from *Schizochytrium* sp. 31. in FM medium.** Figure S4.** GC-FID spectra and area percent report for fatty acid extract from *Schizochytrium* sp. 31. in TM medium.** Figure S5.** GC-FID spectra and area percent report for fatty acid extract from *Schizochytrium* sp. 31. in GM medium.** Figure S6.** GC-FID spectra and area percent report for fatty acid extract from *Schizochytrium* sp. 31. in PPM medium.

## Abbreviations

DHA: docosahexaenoic acid
LC-PUFA: long chain polyunsaturated fatty acids
CM: complex medium
FM: fructose medium
GM: glycerol medium
TM: tryptone medium
PPM: proteose peptone medium
FAME: fatty acid methyl esters
GC: gas chromatography
CM + E: complex medium with ethanol
EPA: eicosapentaenoic acid
