# Supplementary material for: Enhancement of docosahexaenoic acid (DHA) production from Schizochytrium sp. S31 using different growth medium conditions
Source: AMB Express. 2018 Jan 24;8:7. doi: 10.1186/s13568-018-0540-4 (PMC5783985; doi:10.1186/s13568-018-0540-4)
Supplement: Supplementary file 1 — Additional file 1: Figure S1. GC-FID spectra and area percent report for fatty acid extract from Schizochytrium sp. 31. in CM medium. Figure S2. GC-FID spectra and area percent report for fatty acid extract from Schizochytrium sp. 31. in CM+E medium. Figure S3. GC-FID spectra and area percent report for fatty acid extract from Schizochytrium sp. 31. in FM medium. Figure S4. GC-FID spectra and area percent report for fatty acid extract from Schizochytrium sp. 31. in TM medium. Figure S5. GC-FID spectra and area percent report for fatty acid extract from Schizochytrium sp. 31. in GM medium. Figure S6. GC-FID spectra and area percent report for fatty acid extract from Schizochytrium sp. 31. in PPM medium. [file 13568_2018_540_MOESM1_ESM.doc]

**Title:** Enhancement of Docosahexaenoic acid (DHA) Production from *Schizochytrium* sp. S31 Using Different Growth Medium Conditions

**Journal: AMB Express**

**Authors:** Deniz Sahin, Ezgi Tas, Ulkü Hüma Altindag

Department of Molecular Biology and Genetics, Istanbul Technical University, Istanbul, Turkey

***Corresponding author:**

Dr. Deniz Sahin

Address: Istanbul Technical University, Department of Molecular Biology and Genetics, Maslak, 34469, Istanbul, Turkey,

E-mail: [sahinden@itu.edu.tr](mailto:sahinden@itu.edu.tr),

Phone: +905306558226


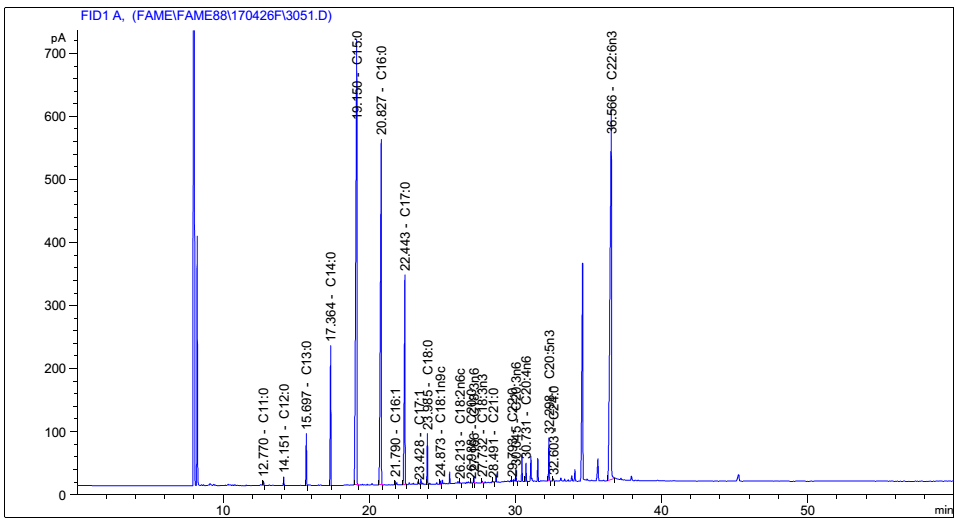


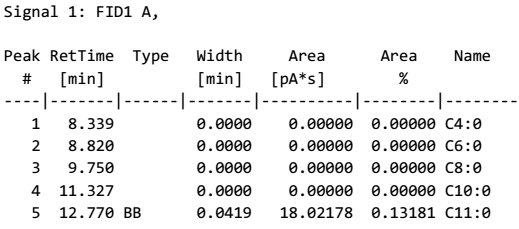


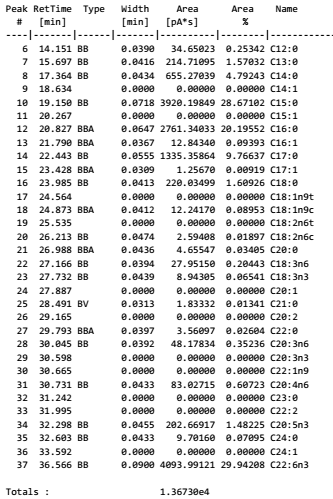


**Fig. AF1** GC-FID spectra and area percent report for fatty acid extract from *Schizochytrium* sp 31. in CM medium.


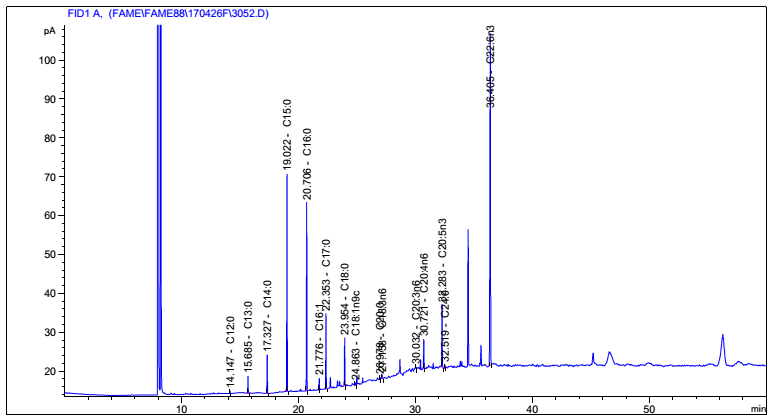


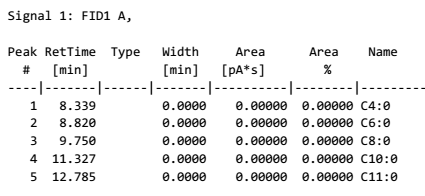


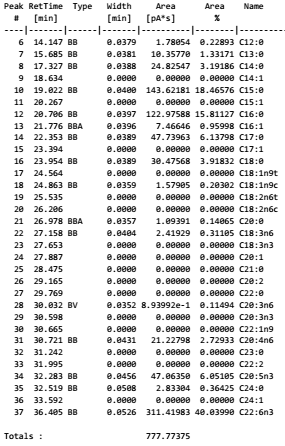


**Fig. AF2** GC-FID spectra and area percent report for fatty acid extract from *Schizochytrium* sp 31. in CM+E medium.


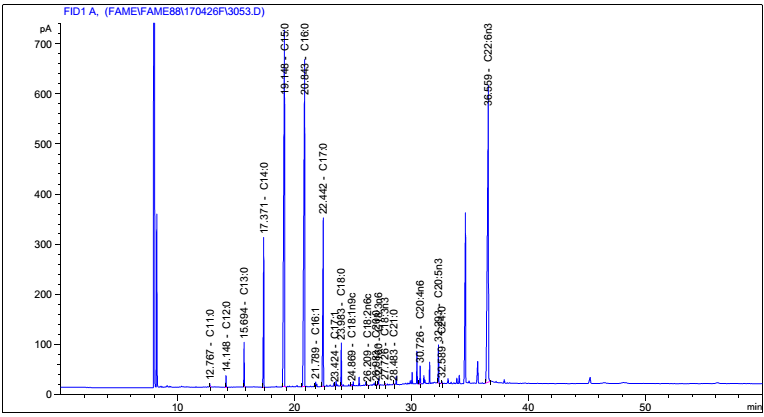


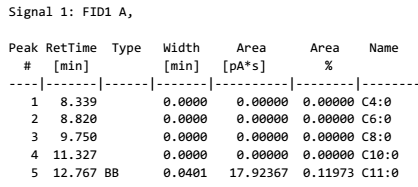


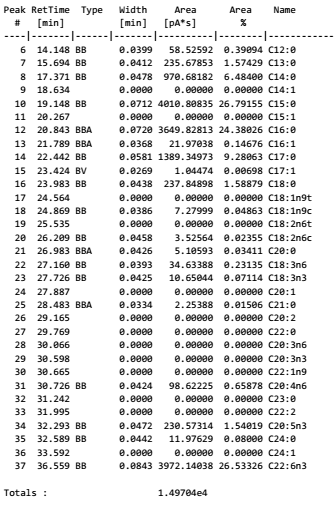


**Fig. AF3** GC-FID spectra and area percent report for fatty acid extract from *Schizochytrium* sp 31. in FM medium.


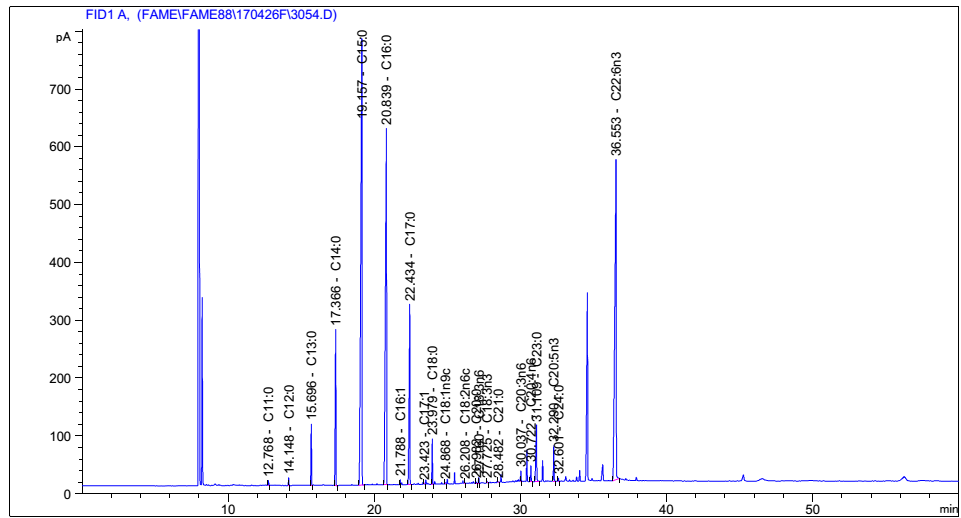


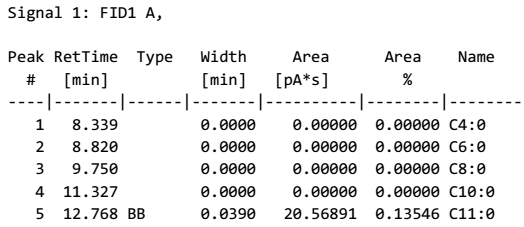


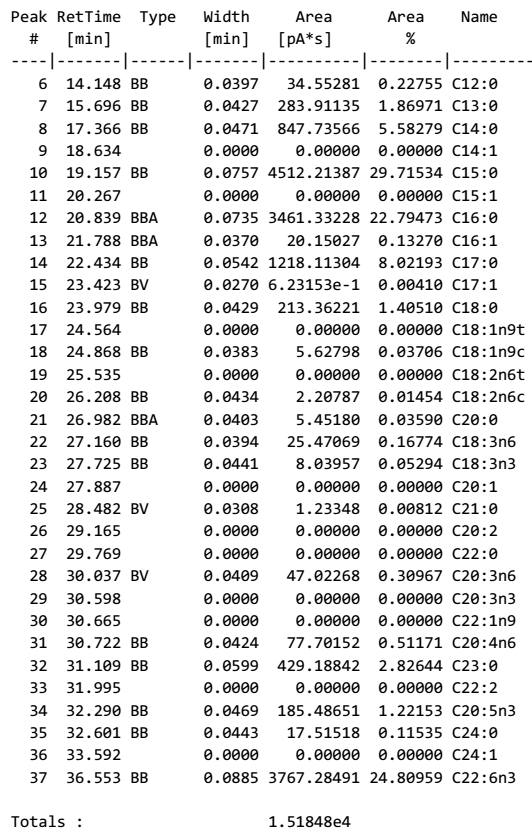


**Fig. AF4** GC-FID spectra and area percent report for fatty acid extract from *Schizochytrium* sp 31. in TM medium.


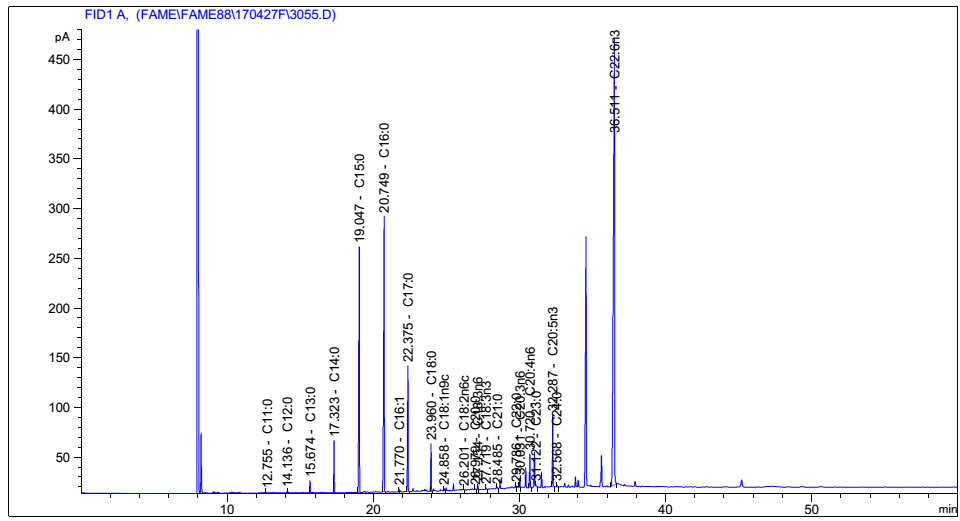


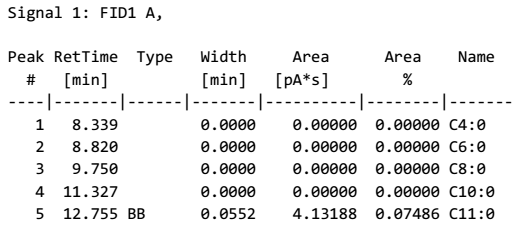


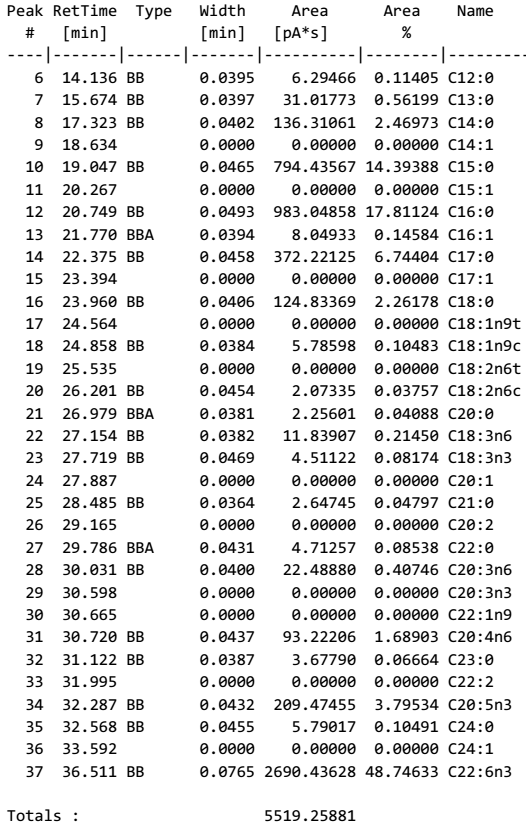


**Fig. AF5** GC-FID spectra and area percent report for fatty acid extract from *Schizochytrium* sp 31. in GM medium.


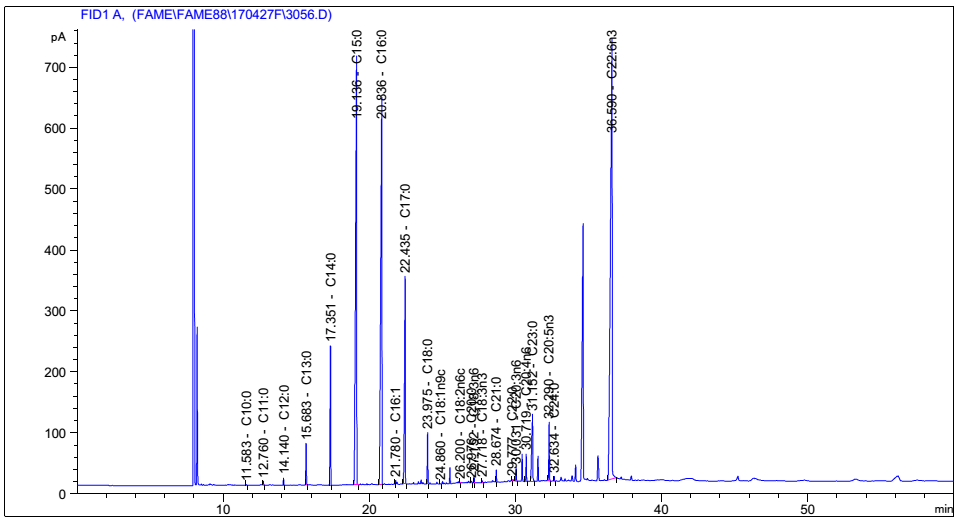


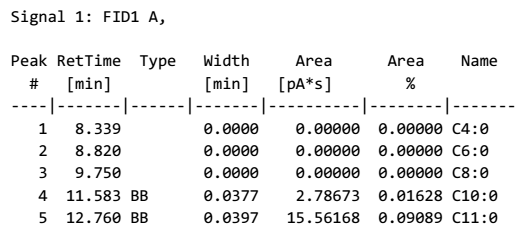


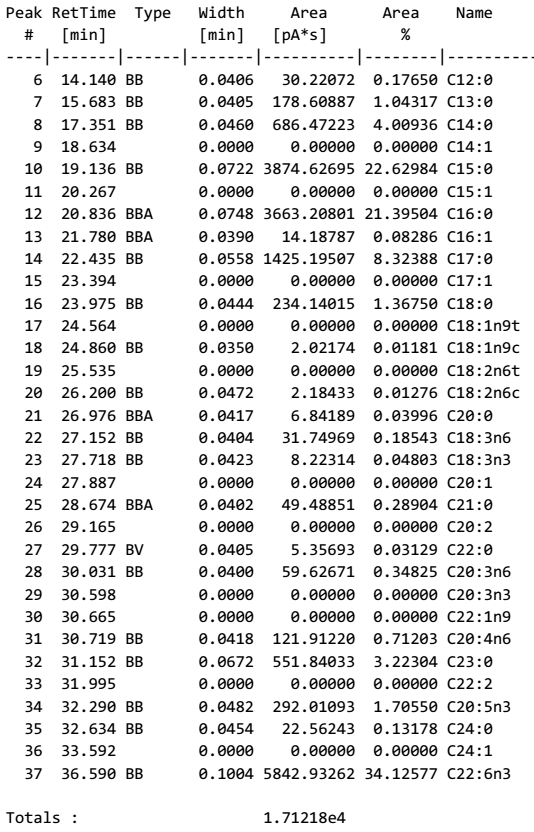


**Fig. AF6** GC-FID spectra and area percent report for fatty acid extract from *Schizochytrium* sp 31. in PPM medium.
